# A Family-Based Study of Inherited Genetic Risk in Lipedema

**DOI:** 10.1089/lrb.2023.0065

**Published:** 2024-04-17

**Authors:** Steven Morgan, Isabella Reid, Charlotte Bendon, Musarat Ishaq, Ramin Shayan, Bernard Pope, Daniel Park, Tara Karnezis

**Affiliations:** ^1^ Lymphatic, Adipose and Regenerative Medicine Group, Department of O'Brien Institute, St Vincent's Institute of Medical Research, Fitzroy, Australia.; ^2^ Department of Plastic and Reconstructive Surgery, West Wing, John Radcliffe Hospital, Headley Way, Oxford, United Kingdom.; ^3^ Department of Medicine, St Vincent's Hospital, Fitzroy, Australia.; ^4^ Melbourne Bioinformatics, The University of Melbourne, Parkville, Australia.; ^5^ Department of Surgery (Royal Melbourne Hospital), The University of Melbourne, Parkville, Australia.; ^6^ Department of Clinical Pathology, The University of Melbourne, Parkville, Australia.; ^7^ Precision Medicine, School of Clinical Sciences at Monash Health, Monash University, Melbourne, Australia.; ^8^ Department of Biochemistry and Pharmacology, The University of Melbourne, Parkville, Australia.

**Keywords:** lipedema, family study, genetic risk

## Abstract

**Background:**

Lipedema is a progressive condition involving excessive deposition of subcutaneous adipose tissue, predominantly in the lower limbs, which severely compromises quality of life. Despite the impact of lipedema, its molecular and genetic bases are poorly understood, making diagnosis and treatment difficult. Historical evaluation of individuals with lipedema indicates a positive family history in 60%–80% of cases; however, genetic investigation of larger family cohorts is required. Here, we report the largest family-based sequencing study to date, aimed at identifying genetic changes that contribute to lipedema.

**Methods and Results:**

DNA samples from 31 individuals from 9 lipedema families were analyzed to reveal genetic variants predicted to alter protein function, yielding candidate variants in 469 genes. We did not identify any individual genes that contained likely disease-causing variants across all participating families. However, gene ontology analysis highlighted vasopressin receptor activity, microfibril binding, and patched binding as statistically significantly overrepresented categories for the set of candidate variants.

**Conclusions:**

Our study suggests that lipedema is not caused by a single exomic genetic factor, providing support for the hypothesis of genetic heterogeneity in the etiology of lipedema. As the largest study of its kind in the lipedema field, the results advance our understanding of the disease and provide a roadmap for future research aimed at improving the lives of those affected by lipedema.

## Introduction

**FIG. 1.**
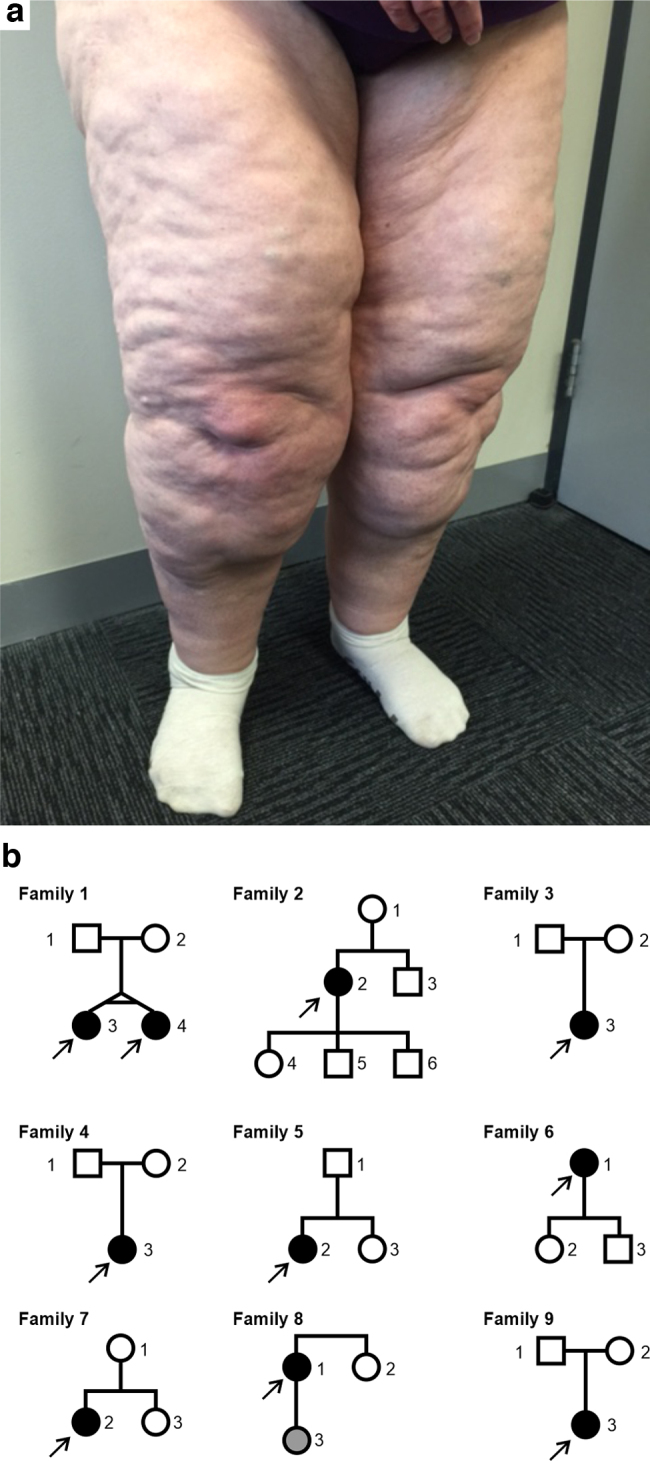
Lipedema phenotype and family pedigrees. **(a)** Typical phenotype of lipedema patient with excessive deposition of subcutaneous adipose tissue in the legs. **(b)** Pedigrees from 9 families with at least one affected lipedema individual in each family. All 31 individuals shown were sequenced. *Arrows* indicate probands. *Square* = male, *circle* = female. Filled shape = diagnosed with lipedema at the time of interview. *Gray shape* = uncertain lipedema status.

Lipedema is a chronic disease characterized by excessive, bilateral, and symmetrical deposition of subcutaneous adipose tissue in the legs, hips, and, less frequently, the arms (Fig. 1a).^1^ It is a progressive condition with symptoms that include severe leg pain, hyperadiposity, swelling, bruising, and hypermobile joint disruption.^1^ Lipedema mostly affects females, with its onset coinciding, typically, with puberty and worsening after periods of hormonal change, such as pregnancy and menopause. Although symptoms first occur between the ages of 10 and 19 years in more than half of patients, individuals seek medical attention on average 17 years after symptom-onset.^1^ An additional 10 years, on average, is required before a lipedema diagnosis is made.^1^ This delay in diagnosis is likely due to poor disease awareness, confusion with obesity or lymphedema, and a lack of known diagnostic biomarkers.^2^ The combination of these factors results in long-term impaired mobility, poor body-image, and significantly impacted quality of life.^3^ In the absence of a cure, current treatments are limited to invasive liposuction, or nonsurgical management strategies to control symptoms or enhance mobility.^4^

The etiology of lipedema is unknown, although genetic inheritance is believed to be a factor, as suggested by patient reports of a positive family history in 60%–80% of cases.^1,5^ Several genes have been implicated in isolated patients or families with lipedema, including *POU1F1A, NSD1,* and *AKR1C1*.^6–8^ Recent studies have used cohorts of multiple unrelated patients to investigate whether there are common genetic factors. In a genomewide association study (GWAS), no single-nucleotide polymorphisms passed the GWAS significance threshold in a cohort of 130 lipedema patients compared to matched controls.^9^ Michelini et al. constructed a panel of 305 genes potentially associated with lipedema, including those identified in previous studies. Three hundred five genes were sequenced and 21 predicted deleterious variants were discovered in 17 out of the 167 patients in the cohort.^10^ Given the current lack of clarity surrounding the genetic basis of lipedema, this multigene panel is an important resource for further genetic analyses.

Given the challenges in diagnosing lipedema, there is a pressing need to understand its genetic basis. We, therefore, sought to generate a large-scale analysis across all genes in families with affected lipedema individuals. We aimed to better understand genetic predisposition to lipedema in affected patients and their unaffected family members.

## Results

### Genetic variants revealed in lipedema families

To characterize the genetics of lipedema, we performed whole-exome sequencing on 31 individuals in 9 families, including 10 probands, 2 of whom were monozygotic twins (Fig. 1b and Supplementary Table S1). In total, 2,786,094 single nucleotide variants (SNVs) and indels were identified using the Genome Analysis Toolkit (GATK) best practices pipeline (11). These were subsequently filtered based on variant quality, inheritance model, gene loss-of-function tolerance, predicted deleteriousness, and population allele frequency (Supplementary Table S2). Results of the variant-calling pipeline and filtering consisted of rare variants (gnomAD global allele frequency <0.01) across 469 genes (Supplementary Fig. S1). Variants were identified in individuals in all 9 families, including 75 that were called in multiple families. No variant was identified in more than four families.

### Gene Ontology analysis of filtered variants

**FIG. 2.**
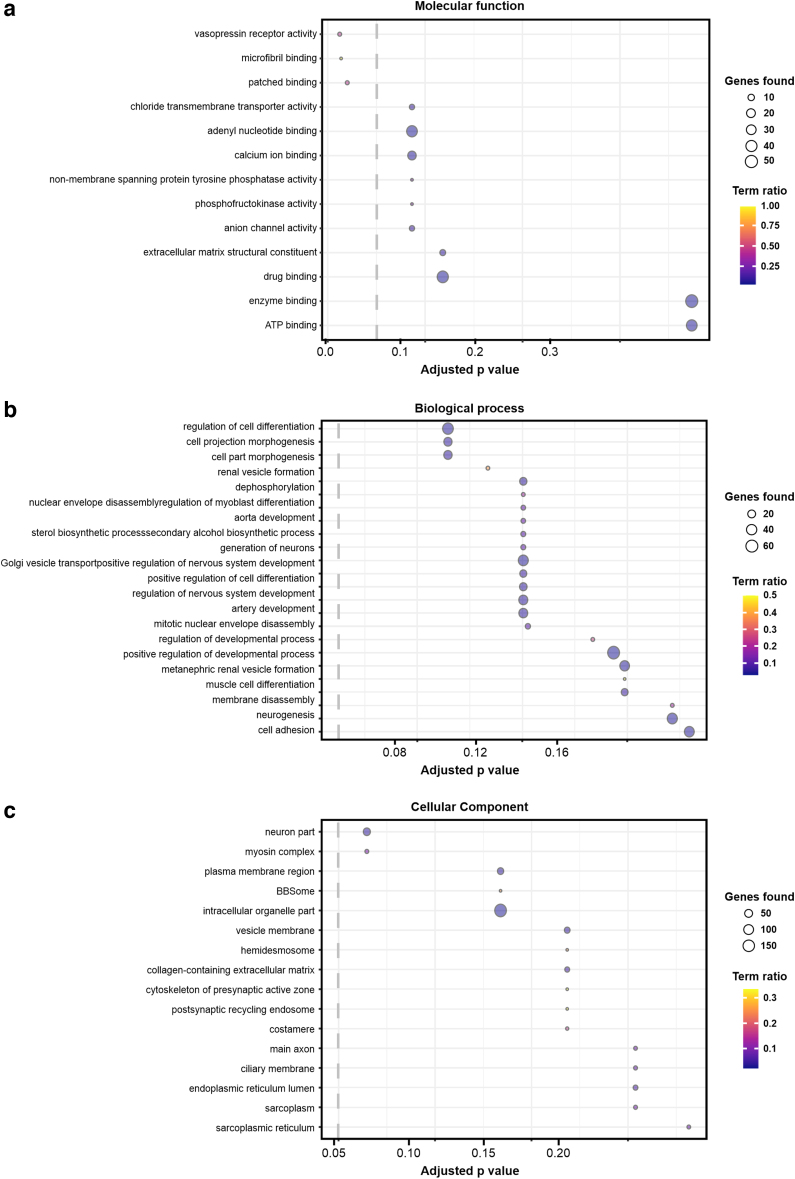
Overrepresented GO terms from filtered variants. The *top* GO terms are shown across the categories of **(a)** molecular function, **(b)** biological process, and **(c)** cellular component. The analysis was conducted using ConsensusPathDB from the total set of candidate variants. Term “ratio” is defined as the number of genes identified as belonging to a particular GO term divided by the total number of genes in that GO term. Adjusted *p*-values are obtained via a hypergeometric test and corrected for multiple hypothesis testing using the false discovery rate method. *Dotted line* shows the statistical significance threshold of Adjusted *p*-value = 0.05. GO, Gene Ontology.

Following variant calling, we applied biochemical pathway analysis applied to all filtered variants using ConsensusPathDB.^11^ This approach identifies overrepresented Gene Ontology (GO) terms via a hypergeometric test. The goal of this test is to determine whether specific biological processes, molecular functions, and cellular components are overrepresented among the genes that harbor the identified genetic variants. In this study, we defined the threshold of statistical significance to be *p* = 0.05, after correcting for multiple hypothesis testing using the false discovery rate method. Three GO terms relating to biological process or cellular component categories passed the significance threshold: vasopressin receptor activity, microfibril binding, and patched binding (Fig. 2).

## Discussion

By recruiting multiple participant families and performing exomewide variant calling, our study aims to fill a knowledge gap by improving the understanding of genetic predisposition to lipedema. Our patient cohort, which includes the first reported case of lipedema-affected monozygotic twins, had SNVs and indels identified across 469 genes. No gene was found to be mutated in every family, suggesting that no single exomic factor is responsible for lipedema in all cases. These results are consistent with the findings of Grigoriadis et al. who suggest that a genetic background, which could include genetic variants in multiple genes, together with environmental factors, confer susceptibility to lipedema.^9^ Taken together, our results and the existing lipedema literature suggest that lipedema is not a simple disease and likely has complex underlying genetic etiology.

A GO analysis of the filtered variant set found overrepresentation of variants related to vasopressin receptor activity, microfibril binding, and patched binding (Fig. 2). These findings provide potential directions for further research into the underlying mechanisms of lipedema pathophysiology. Studies with larger sample sizes with increased statistical power could help to determine if these findings can be replicated in other populations. Notably, the connection between microfibril binding and lipedema is of particular interest, as microfibrils are known to play a crucial role in connective tissue function, and lipedema is a disease that affects connective tissue.^12^

To further contextualize our results, we identified variants in seven of the genes contained in the lipedema panel constructed by Michelini et al.: *BBS1*, *BBS4*, *POMC*, *NCOA1*, *RREB1*, *STAB1*, and *TNXB*. Both our study and the study by Michelini et al. identified genetic variants in the *POMC* gene, which has been linked to obesity predisposition.^10,13^ We identified a missense mutation (NC_000002.12:g.25161709G>A, rs752644128) that was predicted to be deleterious, while Michelini et al. detected a truncating variant (NC_000002.12:g.25161269C>A, rs202127120).^10^ Two of the genes contained in the lipedema panel and identified in our analysis, *STAB1* and *TNXB*, are associated with the extracellular matrix (ECM), in line with the overrepresentation of microfibril binding from the GO analysis.

The largest transcriptomics analysis of lipedema tissue to date found genes related to ECM organization, including genes encoding integrins and collagens, were differentially expressed compared to controls.^14^ Furthermore, spheroid models of adipocytes grown from lipedema adipose-derived stem cells exhibit reduced gene expression of matrix metalloproteinase, key ECM regulators, compared to healthy control spheroids.^15^ We found variants in several ECM-related genes including *STAB1*, which encodes a scavenger receptor that clears collagen-binding proteins in the ECM and has also been associated with waist-to-hip ratio in humans.^16^ We identified a missense variant (NC_000003.12:g.52511686G>A, rs368198386) in family 6 following an autosomal dominant inheritance model. We also detected a *de novo* missense variant (NC_000006.12:g.32056108G>A, rs562786887) in the *TNXB* gene, which encodes the ECM glycoprotein tenascin-X. Deficiency of tenascin-X causes abnormal elastin fiber morphology and reduced collagen levels in the dermis.^17^ Haploinsufficiency of *TNXB* causes the hypermobile type of Ehlers–Danlos syndrome, which is more prevalent in females compared to males.^18,19^ This connective tissue disorder subtype is characterized by joint hypermobility and easy bruising, both symptoms of lipedema.^19^ Our study is the first exomewide analysis to identify ECM-related rare genetic variants in families with lipedema.

Our results suggest that lipedema is likely to be influenced by complex underlying genetic factors. Thus, further studies with increased patient populations, including larger multigenerational families, are needed to reveal the full extent of genetic variation in lipedema. In this study, as in all whole-exome-sequencing based studies, variants that lie in regions of low sequencing depth or outside of the exome are not captured. The investigation of genetic variation in these regions could be performed through whole-genome sequencing. Moreover, larger patient cohorts in future studies could explore the possibility of complex interactions between genetic variants and reduced penetrance in lipedema-affected families. In addition, single-cell sequencing has the potential to significantly enhance our understanding of lipedema through the characterization of tissue heterogeneity and cellular diversity within affected tissue.

## Conclusions

This study, which constitutes the largest family-based sequencing investigation of lipedema to date, provides support for the hypothesis of genetic heterogeneity in the etiology of the disease. Our results demonstrate that lipedema is unlikely to be caused by a single exomic factor across all families. Additionally, the results of our GO analysis suggest that microfibril binding, vasopressin receptor activity, and patched binding may play a role in the disease's development. By providing new insights into the genetic basis for lipedema, this study provides focus for future investigation into this debilitating disease.

## Methods

### Participants

Thirty-one individuals from 9 families were recruited, including 10 probands diagnosed with lipedema. All affected individuals were females and had negative lymphedema status. Two of the probands were monozygotic twins. The criteria described by Wounds UK were used for the diagnoses.^4^ A clinical history was taken via questionnaire for all participants, including family history of lipedema. Pedigree diagrams for all families can be found in Figure 1b. Participants' clinical features are included in Supplementary Table S1.

### Ethics approval and consent to participate

Study protocols (HREC 16 SVHM 38 and HREC 16 SVHM 141_1) were approved by St. Vincent's Hospital, Melbourne, Human Research Ethics Committee, and conducted in accordance with the Declaration of Helsinki.

Written informed consent was obtained from the participants for this publication.

### Variant calling and variant filtering

Whole-exome sequencing was performed on DNA samples extracted from whole venous blood biospecimens for all 31 participants. The GATK best practices pipeline was used to call variants.^20^ Reported sex, ethnicity, and relatedness to other study individuals were confirmed for each sample using Peddy.^21^ Variants were filtered according to variant quality, inheritance model, gene loss-of-function tolerance, predicted deleteriousness, and population allele frequency. Detailed documentation variant filtering is available in the Supplementary Data. The variant calling workflow is illustrated in Supplementary Figure S1.

In the absence of a settled consensus on the prevalence of lipedema, we limited our scope to rare variants, informed by Kobayashi et al., who found that 97% of pathogenic variants in a broad range of clinical areas, with varying inheritance modes and penetrance, had allele frequencies less than 0.0001.^22^ A gnomAD global allele frequency threshold of 0.0001 was used for dominant and *de novo* inheritance models. A threshold of 0.01 was used for recessive and compound heterozygous models. A LoFtool threshold of 0.9 was used, resulting in the removal of variants within genes predicted to be in the highest 10% for loss-of-function tolerance. A Phred scaled Combined Annotation Dependent Depletion (CADD) score of 15 was used as a threshold for predicted deleteriousness. Variants predicted to be of high impact by Ensembl Variant Effect Predictor were retained regardless of CADD score.

Eleven inheritance models were considered (Supplementary Data) including compound heterozygous inheritance where applicable according to Kamphans et al.^23^ No assumptions were made regarding the genotypes or lipedema status of individuals who did not participate in the study. Models allowing male carriers were included because sex limitation has been hypothesized to influence the skewed sex ratio among lipedema patients.^24^ Models that allow male carriers do not make any assumptions regarding the genotypes of unaffected males. To utilize information from unaffected family members, we made the assumption that the lipedema phenotype is fully penetrant, aside from models allowing for male carriers.

### Pathway analysis

ConsensusPathDB was used to identify GO terms that were overrepresented in the total set of filtered variants.^11^ This method uses a hypergeometric test of the provided input genes against the GO database. We used a statistical significance of *p* = 0.05 for this test, after correcting for multiple hypothesis testing using the false discovery rate method.

## Supplementary Material

Supplemental data

Supplemental data

Supplemental data

Supplemental data

## Data Availability

The dataset supporting the conclusions of this article is included in the supplementary files. The genetic variants identified in this study are available in Supplementary Table S2. Other genomic data are available from the corresponding authors on reasonable request.
