# Supplementary material for: A Family-Based Study of Inherited Genetic Risk in Lipedema
Source: Lymphat Res Biol. 2024 Apr 17;22(2):106–11. doi: 10.1089/lrb.2023.0065 (PMC11044871; doi:10.1089/lrb.2023.0065)
Supplement: Supplemental data [file Suppl_TableS1.pdf]

Supplementary Table S1. Participant lipedema status.

Family	Individual	Sex	Lipedema Diagnosis	Lipedema Age of Onset (years)
1	1	M	N	
	2	F	N	
	3	F	Y	14
	4	F	Y	14
2	1	F	N	
	2	F	Y	14
	3	M	N	
	4	F	N	
	5	M	N	
	6	M	N	
3	1	M	N	
	2	F	N	
	3	F	Y	37
4	1	M	N	
	2	F	N	
	3	F	Y	16
5	1	M	N	
	2	F	Y	13
	3	F	N	
6	1	F	Y	15
	2	F	N	
	3	M	N	
7	1	F	N	
	2	F	Y	13-19
	3	F	N	
8	1	F	Y	50
	2	F	N	
	3	F	Uncertain	
9	1	F	N	
	2	F	N	
	3		Y	15-16

Affected individuals are shaded in gray. Age of onset is a best estimate; some probands became aware of the condition in later life.
