# Supplementary material for: A Family-Based Study of Inherited Genetic Risk in Lipedema
Source: Lymphat Res Biol. 2024 Apr 17;22(2):106–11. doi: 10.1089/lrb.2023.0065 (PMC11044871; doi:10.1089/lrb.2023.0065)
Supplement: Supplemental data [file Suppl_FigS1.pdf]

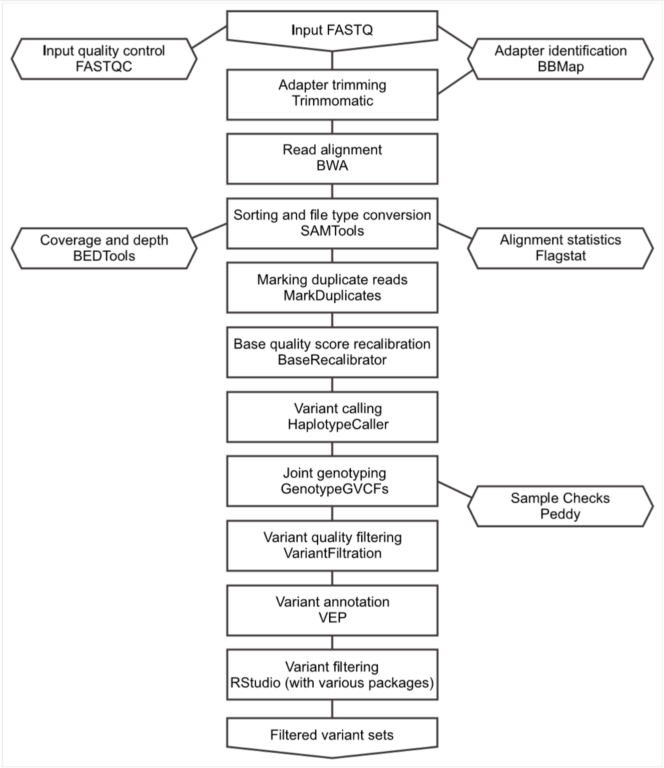

Supplementary Figure S1. **Bioinformatics workflow diagram.**

Bioinformatics steps transform the data from FASTQ files to filtered variant sets. The name of the software tool used is shown below the name of the workflow step. See Supplementary Methods for more details.
