# Supplementary material for: A Family-Based Study of Inherited Genetic Risk in Lipedema
Source: Lymphat Res Biol. 2024 Apr 17;22(2):106–11. doi: 10.1089/lrb.2023.0065 (PMC11044871; doi:10.1089/lrb.2023.0065)
Supplement: Supplemental data [file Suppl_Data.docx]

**Supplementary Methods**

**Variant calling bioinformatics methods**

INPUT QUALITY CONTROL: FastQC was used for quality control of the input FASTQ files.^1^

ADAPTER IDENTIFICATION: The adapter discovery function of BBMap was used in cases where FASTQC identified overrepresented k-mers.^2^

ADAPTER TRIMMING: Trimmomatic was used to remove adapter sequences identified by BBMap. The tool was run in paired-end mode and discarded reads less than 50 bases in length after trimming.^3^

READ ALIGNMENT: BWA-MEM (4) was used to align the reads to the assembly version of GRCh38.^4^

SORTING: SAMtools was used to sort the aligned reads by genome coordinates.^5^

COVERAGE AND DEPTH COMPUTATION: Coverage and depth statistics were computed using BEDTools.^6^ BED files that specify the capture regions were obtained from the exome capture kit manufacturers.

ALIGNMENT STATISTICS: Alignment summary statistics were computed using flagstat from SAMtools.^5^

DUPLICATE MARKING: MarkDuplicates from Picard was used to flag duplicate reads.^7^

BASE QUALITY SCORE RECALIBRATION: BaseRecalibrator was used to adjust the base quality scores in the deduplicated BAM files.^8^ The data used for training the model was a combination of SNVs from dbSNP and indels from the 1000 Genomes Project.^9,10^

VARIANT CALLING: Both SNVs and indels were called per sample by HaplotypeCaller.^11^

JOINT GENOTYPING: The per-sample variants were consolidated into a VCF file and jointly called by GenotypeGVCFs.^8^

SAMPLE CHECKS: Peddy was used to compare the exome data against clinical information that was recorded in participant interviews.^12^ Sex, ethnicity and relatedness to other study individuals were checked.

QUALITY FILTER APPLICATION: Quality filtering was applied using VariantFiltration by GATK.^8^ Filters for statistics related to depth, strand bias, mapping quality and read position were applied separately for SNVs and indels according to the recommendations of the Broad Institute.^13^

VARIANT ANNOTATION: VEP was used to annotate variants.^14^ The Ensembl transcript set was used for transcript-related annotations.

VARIANT FILTERING

Variants were filtered using the annotations applied by VariantFiltration and VEP. Filters were applied per family according to a particular inheritance model. Eleven inheritance models were considered (see below). In order to utilize information from unaffected family members, we made the assumption that the lipedema phenotype is fully penetrant, aside from models allowing for male carriers. The inheritance models utilized were as follows: autosomal recessive, autosomal dominant, X-linked recessive, X-linked dominant, autosomal recessive allowing male carriers, autosomal dominant allowing male carriers, X-linked recessive allowing male carriers, X-linked dominant allowing male carriers, compound heterozygous, compound heterozygous allowing male carriers, and *de novo.*

No assumptions were made regarding the genotypes or lipedema status of individuals who did not participate in the study. The variation in family structures resulted in the same inheritance model being applied differently to different families. For example, where an affected individual and their two unaffected parents were sequenced, compound heterozygous variants were identified according to Kamphans et al.^15^ Where this family structure is absent, a ‘double-hit’ strategy was used. Models that allow male carriers do not make any assumptions regarding the genotypes of unaffected males. In this way, a set of variants from a model allowing male carriers is a superset of the same model without allowing male carriers . The models allowing male carriers were included because sex limitation has been hypothesised to influence the skewed sex ratio among affected individuals.^16^

The following filters were applied to the raw variants to obtain a set of filtered variants. In the absence of a settled consensus on the prevalence of lipedema, we limited our scope to rare variants, informed by Kobayashi et al. who found that 97% of pathogenic variants in a broad range of clinical areas, with varying inheritance modes and penetrance, had allele frequencies less than 0.0001.^17^ Variants were discarded that failed at least one of the quality filters applied using VariantFiltration. Variants that were not located within genes, polymorphic pseudogenes, promotors, transcription factor binding sites or enhancer regions were discarded. Only variants that fit the given inheritance model were retained. A gnomAD global allele frequency threshold was used to retain rare variants. A threshold of 0.0001 was used for dominant and *de novo* models. A threshold of 0.01 was used for recessive and compound heterozygous models. Variants without gnomAD allele frequency information were retained. A LoFtool threshold of 0.9 was used, resulting in the removal of variants within genes predicted to be in the highest 10% for loss-of-function tolerance. Variants in genes without a LoFtool score were retained. Finally, a Phred scaled CADD score of 15 was used as a threshold. Variants without a CADD score were retained. Variants that were predicted to have a high impact by VEP were retained regardless of CADD score.

The filters mentioned above were applied on an per-variant basis. For compound heterozygous models, pairs of variants that passed the above filters were considered on a per-gene basis.

**REFERENCES**

1. Andrews S. FastQC: a quality control tool for high throughput sequence data. 2010. Available from: http://www.bioinformatics.babraham.ac.uk/projects/fastqc. [Last Accessed; 11/5/2023].

2. Bushnell B. BBMap: a fast, accurate, splice-aware aligner. Lawrence Berkeley National Lab.(LBNL), Berkeley, CA (United States): 2014.

3. Bolger AM, Lohse M, Usadel B. Trimmomatic: a flexible trimmer for Illumina sequence data. Bioinformatics 2014;30(15):2114-2120

4. Li H, Durbin R. Fast and accurate short read alignment with Burrows–Wheeler transform. Bioinformatics 2009;25(14):1754-1760

5. Li H., Handsaker B., Wysoker A., Fennell T., et al. The Sequence alignment/map (SAM) format and SAMtools. Bioinformatics 2009;25(16):2078-9

6. Quinlan AR, Hall IM. BEDTools: a flexible suite of utilities for comparing genomic features. Bioinformatics 2010;26(6):841-842

7. Institute TB. Available from: https://broadinstitute.github.io/picard/. [Last Accessed; 11/5/2023].

8. Van der Auwera GA, Carneiro MO, Hartl C, et al. From FastQ data to high‐confidence variant calls: the genome analysis toolkit best practices pipeline. Current protocols in bioinformatics 2013;43(1):11.10. 1-11.10. 33

9. Sherry ST, Ward M-H, Kholodov M, et al. dbSNP: the NCBI database of genetic variation. Nucleic acids research 2001;29(1):308-311

10. Consortium GP. An integrated map of genetic variation from 1,092 human genomes. Nature 2012;491(7422):56

11. Poplin R, Ruano-Rubio V, DePristo MA, et al. Scaling accurate genetic variant discovery to tens of thousands of samples. BioRxiv 2017;201178

12. Pedersen BS, Quinlan AR. Who’s Who? Detecting and resolving sample anomalies in human DNA sequencing studies with peddy. The American Journal of Human Genetics 2017;100(3):406-413

13. Auwera GVd. Apply hard filters to a call set. 2013. Available from: https://gatkforums.broadinstitute.org/gatk/discussion/2806/howto-apply-hard-filters-to-a-call-set [Last Accessed; November 12].

14. McLaren W, Gil L, Hunt SE, et al. The ensembl variant effect predictor. Genome biology 2016;17(1):122

15. Kamphans T, Sabri P, Zhu N, et al. Filtering for compound heterozygous sequence variants in non-consanguineous pedigrees. PloS one 2013;8(8):e70151

16. Child AH, Gordon KD, Sharpe P, et al. Lipedema: an inherited condition. American Journal of Medical Genetics Part A 2010;152(4):970-976

17. Kobayashi Y, Yang S, Nykamp K, et al. Pathogenic variant burden in the ExAC database: an empirical approach to evaluating population data for clinical variant interpretation. Genome medicine 2017;9(1):13
